# Better influenza vaccines: an industry perspective

**DOI:** 10.1186/s12929-020-0626-6

**Published:** 2020-02-14

**Authors:** Juine-Ruey Chen, Yo-Min Liu, Yung-Chieh Tseng, Che Ma

**Affiliations:** 1 RuenHuei Biopharmaceuticals, Inc., Taipei, 100 Taiwan; 2 Genomics Research Center, Academia Sinica, Taipei, 115 Taiwan; 3 Institute of Microbiology and Immunology, National Yang Ming University, Taipei, 112 Taiwan

**Keywords:** Influenza virus, Universal vaccine, Monoglycosylated HA, Monoglycosylated split vaccine

## Abstract

Vaccination is the most effective measure at preventing influenza virus infections. However, current seasonal influenza vaccines are only protective against closely matched circulating strains. Even with extensive monitoring and annual reformulation our efforts remain one step behind the rapidly evolving virus, often resulting in mismatches and low vaccine effectiveness. Fortunately, many next-generation influenza vaccines are currently in development, utilizing an array of innovative techniques to shorten production time and increase the breadth of protection. This review summarizes the production methods of current vaccines, recent advances that have been made in influenza vaccine research, and highlights potential challenges that are yet to be overcome. Special emphasis is put on the potential role of glycoengineering in influenza vaccine development, and the advantages of removing the glycan shield on influenza surface antigens to increase vaccine immunogenicity. The potential for future development of these novel influenza vaccine candidates is discussed from an industry perspective.

## Background

Seasonal influenza outbreaks cause 3 to 5 million cases of severe illness and 290,000 to 650,000 respiratory deaths each year [1, 2]. The *Orthomyxoviridae* are a family of enveloped viruses with a genome consisting of 6~8 segments of negative-sense single-stranded RNA, including four genera of influenza virus: A, B, C and D [3]. Influenza A and B are the main cause of annual flu outbreaks in humans, with influenza A further classified into subtypes based on their surface glycoproteins hemagglutinin (HA) and neuraminidase (NA). 18 HA subtypes (H1~H18) and 11 NA subtypes (N1~N11) are currently known, most notable today are the H1N1 and H3N2 subtypes that co-circulate in the human population. Since the 1970s influenza B has diverged into two lineages based on antigenicity, the Yamagata and Victoria lineages, with little or no serum cross-reactivity [4]. In contrast to the severity and epidemic potential of influenza A and B, influenza C infections induce only mild flu symptoms in children, while influenza D is not known to infect humans [5].

Recurrent influenza epidemics with pre-existing immunity occurs because the influenza virus employs two mechanisms to escape recognition: antigenic drift and antigenic shift. Antigenic drift is the gradual accumulation of point mutations on the influenza virus’ surface glycoproteins HA and NA, driven by high error rates (estimated at 1.5 × 10^− 5^ per nucleotide per replication [6]) of the virus’ RNA-dependent RNA polymerase (RdRP). Mutations that allow the virus to evade the host immune system are positively selected for and become fixed, resulting in the rise of new strains that are antigenically different from what the host was vaccinated against. The second escape mechanism, antigenic shift, is the reassortment of gene segments across different strains infecting the same host, resulting in a wholesale change in antigenicity [7, 8]. Antigenic shift have historically been associated with influenza pandemics, the most recent example being the 2009 swine-origin H1N1 that included segments from classical swine H1N1, Eurasian swine H1N1, and a triple reassortant from 1998 [9]. The rise of new strains through antigenic drift and shift is followed by cross-immunity mediated competition between antigenically similar strains, which results in a progressive replacement of existing strains with new variants [10, 11].

Unfortunately, current seasonal influenza vaccines are strain-specific and have a very narrow range of coverage, meaning extensive surveillance, accurate predictions and annual vaccination are needed as circulating strains evolve continuously over time. This task is coordinated by the World Health Organization (WHO) Global Influenza Surveillance and Response System (GISRS), which gathers year-round data from hundreds of national influenza centers around the world and issue vaccine formulation recommendations for each upcoming flu season [12]. When vaccine strains are well-matched with circulating strains, vaccination provides healthy adults younger than 65 years with 70–90% protection [13], and reduced hospitalizations in the elderly and those with chronic illnesses by 30–70% [14–16]. However, in years when there is a mismatch between the vaccine and circulating strains, the vaccine effectiveness (VE) tends to be much lower [17].

Here we discuss various challenges the current seasonal flu vaccine is facing, and how a universal influenza vaccine approach through carbohydrate design to elicit broadly neutralizing antibodies (bnAbs) targeting the influenza HA glycoprotein can potentially play a role in the future of influenza prevention. Despite the first influenza vaccine being commercially available as early as 1945, influenza outbreaks continue to be a major public health concern today. It is imperative for health authorities, researchers and the pharmaceutical industry to work together on improving the efficacy of influenza vaccines.

## Limitations and drawbacks of current influenza vaccines

Traditional trivalent influenza vaccines include two inactivated influenza A strains (H1N1 and H3N2) and one influenza B strain, but this has recently been overtaken by quadrivalent influenza vaccine comprised of H1N1, H3N2 and both influenza B lineages that offers a more complete coverage [18]. Commercially available vaccine options include egg- or cell-based inactivated influenza vaccine (IIV), a live attenuated influenza vaccine (LAIV), and a recombinant HA vaccine produced in insect cells [16].

### Egg-based inactivated influenza vaccines

The production of egg-based influenza vaccines has remained virtually unchanged since the advent of split (subvirion) vaccines in the 1970s, and still commands 88% of the global market share in 2018 [19]. The main advantages of the egg-based platform include an excellent production capacity that is capable of producing an estimated 1.5 billion doses annually, and a low production cost that allows global access to the vaccine [20].

**Fig. 1:**
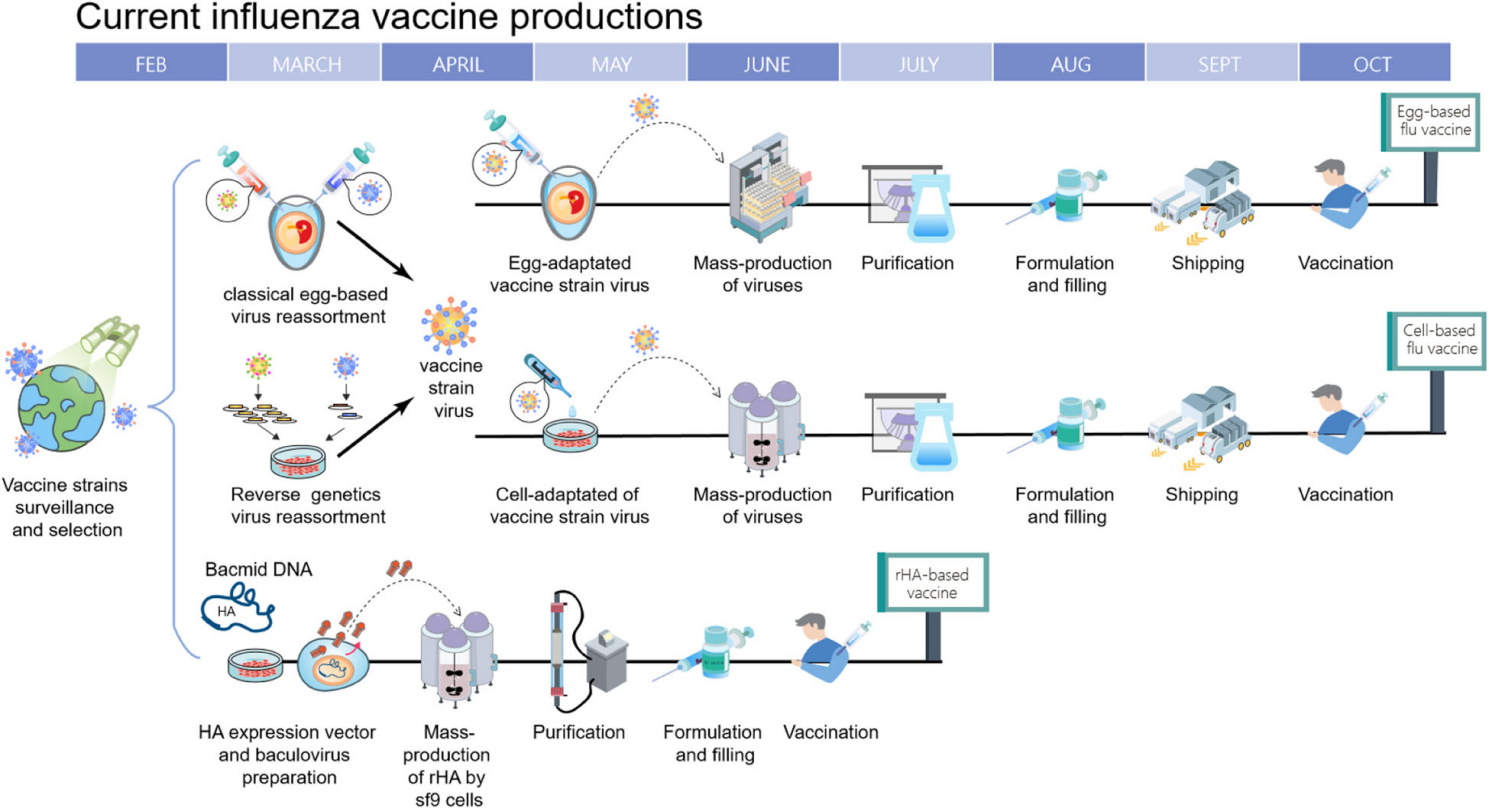
Timeline of current influenza vaccine production methods. Schematic overview of egg-based, cell-based and protein-based influenza vaccine production. Vaccine strains that match circulating influenza viruses for the upcoming flu season are selected by the World Health Organization (WHO) Global Influenza Surveillance and Response System (GISRS). High yielding vaccine strains for egg- or cell-based production are generated by either classic or reverse genetic reassortment. These adapted viruses go into mass production, either in embryonated chicken eggs or MDCK cells with a production timeline of approximately six to eight months. In recombinant HA (rHA) vaccines, the HA sequence is cloned into baculovirus and expressed by insect cells, significantly shortening production time

The strain-specific nature of current vaccines necessitates the annual selection of candidate vaccine viruses (CVVs), including screening the antigenicity of isolates, preparing reassortant viruses, and adaptation of the virus to eggs (Fig. 1). For egg-based manufacture, the entire process from strain selection to vaccine availability typically takes 6~8 months with tight time constraints, and any unexpected circumstance such as a delayed WHO strain recommendation [21] or unexpected low virus yield [22], can snowball into significant production delays and directly affect vaccine supply. This lengthy interval also gives circulating influenza viruses time to mutate, as it did during the 2014–2015 flu season when late-emerging H3N2 variants rendered the recommended vaccine strain ineffective [8].

A second drawback of using an egg-based platform stems from the adaptation process of culturing a human virus in avian tissue, where adaptive mutations may accumulate and potentially change the strain’s antigenicity [23–25]. HA, apart from being the primary target for neutralizing antibodies, is the main facilitator of influenza virus entry by binding to sialic acids on the surface of the host cells. Human influenza HA preferentially bind to α-2,6 linked sialic acids commonly found on epithelial cells in the human upper respiratory tract [26, 27]. However, in egg-based production vaccine strains are inoculated into the allantoic cavity of embryonated chicken eggs which only contain α-2,3 linkages [28]. With successive passages, this becomes a selective pressure that can cause the acquisition or a total shift in receptor specificity, with its accompanying mutations and antigenic changes on HA’s receptor binding site. A recent example of this occurred during the 2016–2017 flu season, when egg-adapted vaccine strains were found to lack a glycosylation site (T160, H3 numbering) on H3N2 HA antigenic site B, one of the five major antigenic sites that induce neutralizing antibodies [24].

A third concern is the egg-based platform relies on a steady supply of embryonated eggs. This egg supply can be overwhelmed by sudden increases in demand, such as during a pandemic.

### Live attenuated influenza vaccines

LAIV is generated by combining the HA and NA of currently circulating strains with the internal proteins of an attenuated cold-adapted strain. This results in a reassortant vaccine virus that can be administered intranasally and has some limited replicative ability in the human upper respiratory tract. As the entire influenza replication cycle is utilized at the site of infection, LAIV has also been reported to elicit cell-mediated immunity [29] and local mucosal immunity [30] besides the induction of a robust antibody response. Clinically, LAIV has shown variable but overall comparable efficacy to IIV in adults and better efficacy in children.

Recently however, the necessity of effective replication in human respiratory tissue has emerged as an area of concern. The US Advisory Committee on Immunization Practices (ACIP) recommended against LAIV between 2016 to 2018 due to low efficacy of the H1N1 component [31], although this phenomenon was not noted in Europe and Canada [32]. The reason for this lack of efficacy is still unclear, but possibilities include viral interference of tetravalent vaccine strains resulting in reduced virus shedding for the weakest strain, strong cross-reactive antibodies from previous seasons preventing virus replication, and inherent lower replication in host tissue by the H1N1 pandemic strain [33], among others. ACIP has since resumed recommendation for LAIV in 2018 following a change in the H1N1 vaccine component [34].

Secondly, as currently-available LAIV is also produced in embryonated chicken eggs, it is plagued by many of the same concerns as egg-based IIV. In 2019 AstraZeneca’s LAIV product FluMist experienced manufacturing issues due to low yields in two strains, resulting in a reduction in shipments worldwide [35].

### Cell-based and recombinant HA vaccines

In order to overcome limitations of the egg-based manufacturing process, production systems using mammalian or insect cell cultures have emerged [36, 37].

The manufacturing process for cell-based IIV is similar to egg-based IIVs, but has several advantages over the latter (Fig. 1). Viral production in a cell culture bioreactor is more flexible, more scalable and unaffected by egg shortages. Additionally, recent comparisons have shown that cell-based vaccines provided a moderately higher VE for elderly individuals (≧65 years old) than egg-based vaccines, possibly due to less egg-adapted mutations [38].

For recombinant HA production in insect cells, the baculovirus expression system is utilized to manufacture recombinant HA, which is then purified and formulated into HA trimer “rosettes” [39]. This not only has the same benefits of speed, flexibility and scalability as cell-based IIV, but also eliminates the reliance on influenza virus replication for vaccine production and the time-consuming process of strain selection. FluBlok, a recombinant HA vaccine developed by Sanofi Pasteur, was found to be 30% more efficacious than traditional IIV for people ≧50 years old [40].

However, the comparatively high cost of these alternatives to egg-based influenza vaccines have prevented them from taking a bigger share of the influenza vaccine market. According to the US Centers for Disease Control (CDC) adult influenza vaccine contract pricing for 2019–2020, the cost of the cell-based vaccine Flucelvax is approximately 40% higher than an inactivated egg-based vaccine produced by the same manufacturer. The recombinant HA vaccine Flublok can be more than twice as expensive as egg-based vaccines [41]. Additionally, while cell-based and recombinant vaccines have the benefit of speed and flexibility that is critical for pandemic preparedness, it does not translate to a competitive advantage on the seasonal vaccine market [42]. So far slow progress has been made to transition away from egg-based production, and more support from governments around the world is needed.

## Next-generation influenza vaccines

Various next-generation influenza vaccines under development aims to broaden or lengthen the human immune response with novel antigens and adjuvants, gradually expanding the strain-specific nature of current vaccines to include all strains within a subtype (eg all H1 strains), multiple subtypes (eg H1/H5/H9), or incorporating all subtypes within a group (influenza A group 1 or group 2), with the ultimate goal of creating a truly “universal” pan-influenza vaccine that can elicit lifelong immunity against all influenza A and B viruses [43].

From a public health perspective influenza continues to be the only human disease that requires annual vaccination. It is estimated that replacing just 10% of seasonal vaccines with a universal vaccine would avert 6300 influenza-related deaths and save 1.1 billion US dollars in direct healthcare costs per year in the United States alone [44]. In 2017, the National Institute of Allergy and Infectious Diseases (NIAID) in the US laid out a detailed strategic plan for the development of a universal influenza vaccine, highlighting knowledge gaps and research areas in pursuit of this common goal [43]. In their outline, they established four criteria for a universal influenza vaccine as: **75% effectiveness against symptomatic influenza infection, protection against both group I and group II influenza viruses, durable protection that last at least 1 year, and be suitable for all age groups.** It is with these criteria in mind that we discuss various vaccine candidates being developed (Table 1).

**Table 1:** Vaccine Candidates Currently Being Developed

Category	Sponsor/ company	Strategy	Phase	Mechanism and potency assay	Reference
HA protein-based vaccine	Novavax, Inc.	HA Rosettes, HA nanoparticles, VLP with Matrix-MTM adjuvant	I/II	Particle format for potency, multiple strains mixed or sequential delivery; HAI and MN assay	[45–47]
	NIH, GSK, and Icahn School of Medicine at Mount Sinai	HA stem or head-stem chimera	I	bnAbs (no HAI) and ADCC; intranasal influenza challenge	[48–51]
	Academia Sinica and OPKO	Monoglycosylated HA as universal flu vaccine, exposing the conserved domain to elicit bnAbs	preclinical	Broad cross-reactive Ab; HAI and MN assay	[52–54]
Epitope-peptides based vaccine	BiondVax Pharmaceuticals Ltd	HA, NP, M1 peptides	II/III	Cytotoxic T lymphocytes (CTL) response	[55–58]
	PepTcell.Ltd	FLU-V	II	Cross-reactive T-cell responses, and mucosal immunity; intranasal influenza challenge	[59–63]
Live attenuated virus vaccine	CodageniX	CodaVax Live-attenuated and single-round whole virus	I/II	Additional antigens, T cell responses, and mucosal immunity; intranasal influenza challenge	[64–66]
	FluGen	M2SR	I/II	T cell responses, and mucosal immunity; intranasal influenza challenge	[67, 68]
DNA based vaccine	Inovio	RNA, DNA, or vector subunit delivery	I	Gene delivery for CTL and Ab	[69–72]
M2-based protein vaccine	Acambis/Sanofi Pasteur	M2 ectodomain	I/II	bnAbs; ADCC (no NT); intranasal influenza challenge	[73, 74]

### Altering glycan composition on recombinant HA and split virus vaccines

Historically, a crucial strategy of influenza virus’ escape from pre-existing immunity is the addition of N-glycosylation sites on the immunodominant HA head domain [75]. These bulky but poorly-immunogenic N-glycans allow the virus to hide antigenically-conserved domains from host immune system recognition [76], a mechanism known as “glycan shielding”.

When H1N1 first emerged in 1918, it carried only one conserved glycosylation site at position 104 (H1 numbering) on the HA head. But as the virus continued circulating in the human population up to the 1950s, it sequentially acquired glycans at positions 144, 172 and 177, all at or adjacent to the major antigenic site Sa on the HA head. This was followed by a 20-year hiatus as H1N1 was supplanted by H2N2, before re-emerging in 1977 carrying the same three acquired and one conserved glycosylation sites as before. The following decades saw N144 replaced by N142, the disappearance of N172, and the acquisition of N71 before the glycan shield was ultimately reset due to the emergence of 2009 pandemic H1N1, carrying only the original conserved glycosylation site on 104 [77]. Conversely, H3N2 circulated in 1968 carrying two glycans on its HA head, N81 and N165 (H3 numbering). Although the glycosylation site at position 81 was subsequently lost, positions 63, 122, 126, 133, 144, and 246 were accrued and retained [78]. Overall, the continued circulation of an influenza subtype in the human population corresponds to a steady increase in N-glycans on its HA head domain. Evidence that these acquired N-glycans provide a shielding effect comes from not only the observation that they tend to appear on or near major antigenic sites, but also studies have shown the acquisition of sites 177 and 142 on H1N1 slow genetic drift in the shielded areas [79], and mutational deletion of 177, 142 and 71 on a pre-pandemic H1N1 strain elicited a protective immune response against the 2009 pandemic H1N1 strain [77]. Similarly, in H3N2 positive selection disappeared when an antigenic site becomes shielded by N-glycans [78], and the introduction of five recent glycosylation sites at positions 63, 122, 126, 133 and 246 allowed a 1968 H3N2 strain to evade polyclonal human serum raised against it [80].

These observations indicate that exposing the comparatively conserved, glycan-shielded regions of viral hemagglutinin could be a potential strategy to increase the breadth of influenza vaccine protection [52, 81, 82]. However, previous attempts have shown complete de-glycosylation of all carbohydrate moieties on influenza HA by either prokaryotic production [52], tunicamycin treatment [83] or PNGase F digestion [53] does not appear to be a viable strategy. Conserved N-glycosylation sites on the HA stem are essential for intracellular transport, correct glycoprotein folding and HA trimerization [84], and a completely unglycosylated HA would have a high chance of altered antigenicity.

Therefore, our group focuses on harnessing glycoengineering techniques to alter N-glycan composition on the HA, creating recombinant HAs that retain only a single N-acetylglucosamine (GlcNAc) attached to asparagine per N-glycosylation site (monoglycosylated HA, or HA_mg_). To accomplish this, N-acetylglucosaminyltransferase I deficient (GnTI^−^) human embryonic kidney cells that are unable to synthesize complex type N-glycans were used to produce secreted, transmembrane domain truncated HAs that have only high mannose residues on their N-glycosylation sites. These high mannose HAs were then further trimmed with the high mannose-cleaving enzyme endoglycosidase H leaving a single GlcNAc residue, dramatically decreasing the size and shielding effect of these N-glycans while still maintaining the native HA structure in its trimeric state.

**Fig. 2:**
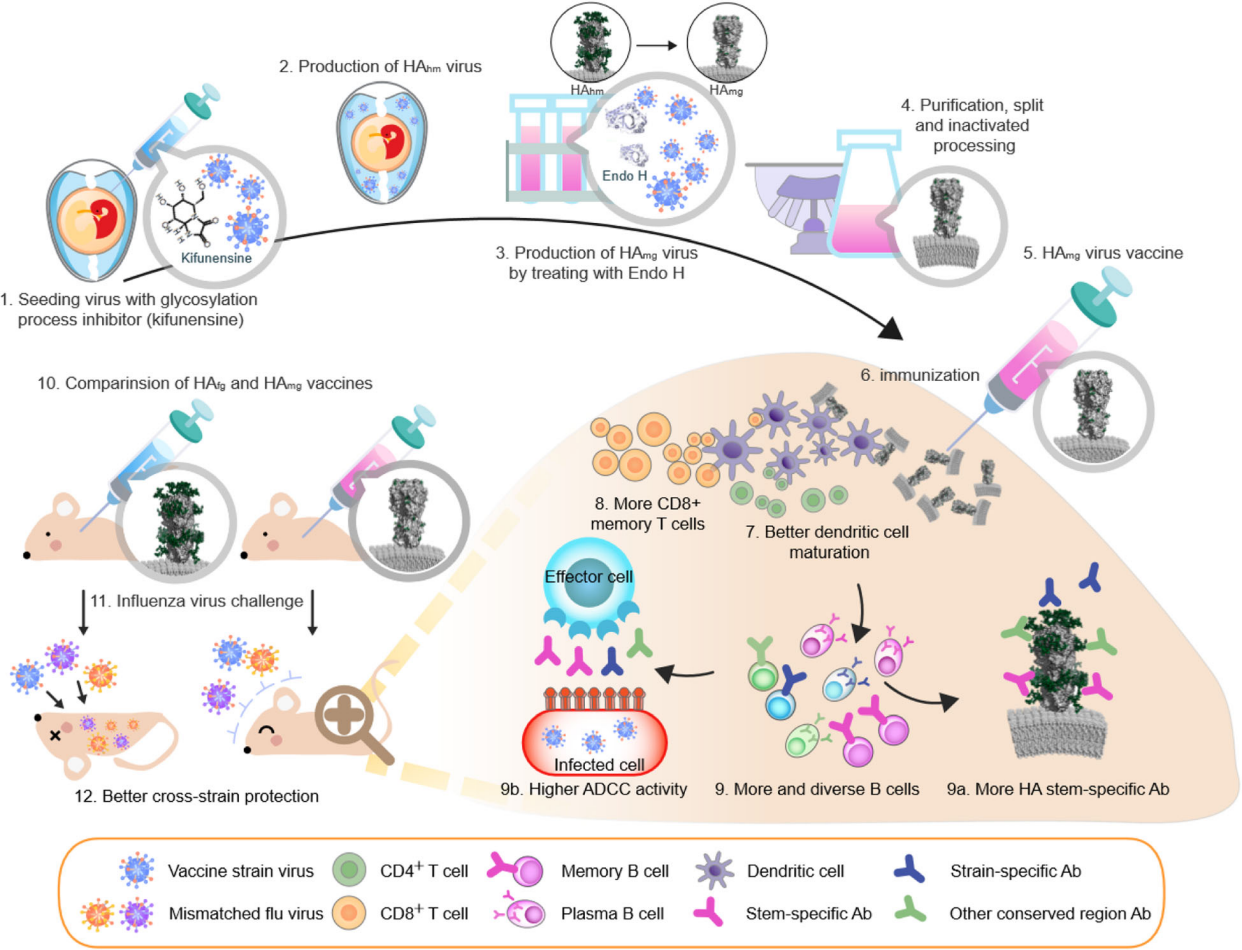
The production and immune response of monoglycosylated influenza vaccine. The production of monoglycosylated split virus vaccine adds two key steps to the traditional egg-based platform. Kifunensine, a mannosidase I inhibitor, is added during egg inoculation to arrest viral glycoprotein processing, resulting in a uniformly high mannose composition. Endoglycosidase H is added after harvest to trim high mannose residues down to a single GlcNAc. The resultant monoglycosylated split vaccine provides a more diverse immune response and more effective cross-strain protection than conventional egg-based vaccines. HA_fg_, non-modified egg-based vaccine with complex type N-glycans attached to HA; HA_hm_, HA with only high mannose type N-glycans; HA_mg_, HA with a single GlcNAc at its N-glycosylation sites. Models of HA_fg_, HA_hm_ and HA_mg_ are created with Protein Data Bank ID code 3LZG and 6FYT by adding glycan with GlyProt (http://www.glycosciences.de/modeling/glyprot/php/main.php), coot (https://www2.mrc-lmb.cam.ac.uk/personal/pemsley/coot/) and PDB of lipid bilayer from Lipid Bilayer Membranes for RasMol (https://www.umass.edu/microbio/rasmol/bilayers.htm). The images were displayed with program PyMOL (www.pymol.org)

Antibodies raised against HA_mg_ inoculation demonstrated better binding affinity, neutralization and cross-reactivity than the unprocessed HA (fully glycosylated HA, or HA_fg_) [52, 53]. HA_mg_ also induced the maturation of dendritic cells, more splenic granzyme B-secreting CD8^+^ T cells, and elicited a more diverse HA-specific B-cell repertoire than that of HA_fg_ when used as a vaccine (Fig. 2). In terms of cross-protection, inoculation with an H1N1 pre-pandemic Bris/07 HA_mg_ not only provided better protection in mice against laboratory strains WSN and PR8, but also offered 70% protection against a pandemic strain [52, 53].

While a recombinant HA_mg_ vaccine would have all the advantages of a cell culture production system including speed, flexibility and safety, egg-based production remains the mainstay of influenza vaccine manufacture today. Devising a simple method to apply the monoglycosylation concept to egg-based vaccines with minimal modification will allow this procedure to be integrated into established production methods. Extensive testing found that kifunensine, an α-mannosidase I inhibitor, can be injected into embryonated eggs to convert influenza virus membrane glycoproteins to a uniformly high mannose composition. After harvesting these virions their high mannose N-glycans were then trimmed with endoglycosidase H to create intact monoglycosylated virus particles, and all participating reagents are removed in subsequent purification steps [54].

Like the recombinant HA_mg_ before, monoglycosylated split inactivated influenza vaccines produced by kifunensine and endoglycosidase H treatment were shown to have higher neutralization and cross-neutralization activity, higher hemagglutination inhibition (HAI), more HA stem selectivity, and higher antibody dependent cellular cytotoxicity (ADCC) (Fig. 2). A monoglycosylated pandemic H1N1 split virus vaccine offered cross-protection against strains as diverse as the pre-pandemic NC/99 and the laboratory strain WSN [54]. Aside from having simplified glycans, this procedure produces antigens that are virtually identical to the current influenza vaccine, and would presumably offer a similar safety profile.

### Recombinant HA vaccines

An adjuvanted recombinant HA trivalent nanoparticle influenza vaccine (tNIV) has been developed by Novavax using the baculovirus expression system to produce recombinant HAs, which were then purified and mixed with polysorbate 80 to form protein-detergent nanoparticles of 2~7 HA trimers [45]. The administration of this tNIV with a saponin adjuvant (Matrix-M) in ferrets induced higher levels of neutralizing antibodies against a panel of A (H3N2) strains than a commercial inactivated vaccine (trivalent Fluzone). A Phase I/II clinical trial showed similar results in patients, where tNIV induced significantly greater HAI responses compared to trivalent Fluzone against not only previous strains, but a forward-drifted A/Singapore variant [46].

Another candidate is a chimeric HA (cHA) vaccine born from a collaboration between Icahn School of Medicine at Mount Sinai and GSK/NIH. This strategy originates from the observation that our immune system tends to focus on the immunodominant but highly variable HA head domain, while the subdominant conserved stem region has a better ability to elicit bnAbs. By sequential immunization with a cHA protein consisting of a stem from circulating strains coupled to an irrelevant HA head from exotic influenzas, the strategy is devised to re-direct our immune system to better stimulate stem-specific responses [48]. In a preclinical study, ferrets sequentially immunized with heterologous influenza strains including live attenuated influenza vaccine (LAIV) bearing an H8 head domain and an H1 stem domain (cH8/1) and a split-inactivated vaccine bearing an H5 head domain and an H1 stem domain (cH5/1), conferred superior protection against challenge with pandemic H1N1 virus following different prime-boost combinations and immunization regimens [49]. This approach is currently in collaboration with GSK in a phase I study, and clinical data will be obtained by the end of 2019.

### Epitope-peptide based vaccines

Multimeric-001 (M-001) is a vaccine currently being developed by BiondVax Pharmaceuticals consisting of nine conserved B and T cell epitopes from HA, nucleoprotein (NP) and matrix 1 (M1) protein arranged in triplicate and put onto a single recombinant protein [57]*.* Phase I/II clinical studies have shown the M-001 vaccine induced both cellular and humoral immunity to influenza A and B strains as a standalone vaccine [58], and also enhanced seroconversion when used as a primer for elderly patients before inoculation with inactivated trivalent vaccines [85].

FLU-v is another epitope-based vaccine developed by SEEK (PepTcell) based on in silico multiple alignment of influenza sequences and prediction of possible T-cell epitopes. Six consensus sequences from influenza NP, M1 and matrix 2 (M2) proteins were identified and synthesized into a candidate vaccine. Flu-v has been shown to induce a specific CD8^+^ response against these conserved epitopes and confer protection against heterotypic infection in mice [59], and a Phase Ib challenge trial also showed that the blood cells from immunized subjects exhibited cross reactive immunity against different influenza viruses [62, 63].

### Live attenuated influenza vaccines

CodaVax is an LAIV being developed by Codagenix that takes advantage of inherent human codon pair bias to reconstruct the influenza viral genome with synonymous but sub-optimal codons. This results in viral proteins that have the same amino acid sequence and antigenicity as wild type strains but attenuated due to excessive use of rare codons [64, 65]. In animal models, the vaccine is shown to be effective at lower doses than conventional LAIV [66]. CodaVax has scheduled a phase I/II trial in the first quarter of 2017.

M2SR is an M2 deficient single-replication LAIV being developed by FluGen. In this strategy the M2 sequence in the viral genome (critical for viral uncoating and assembly) is largely deleted, but viruses are produced in M2-expressing cells to generate infective virions. Therefore, after inoculation into a host the attenuated virus is unable to propagate infective progeny, limiting the infection to a single round of replication [67]. In a ferret model M2SR was found to be less susceptible to the negative effects of pre-existing immunity on drifted strains [68]. Initial results from a placebo-controlled phase II trial indicate that the vaccine was effective against a mismatched H3N2 challenge.

### DNA-based vaccine

Inovio has made efforts to apply their Syncon® synthetic DNA vaccine platform to influenza. By sequence alignment and cluster grouping of HA they have generated four “micro-consensus” sequences within an influenza subtype, which were then cloned onto expression vectors and delivered to the vaccine recipient via in vivo electroporation [72]. In mouse and ferret models these micro-consensus sequences against H1N1, H3N2 and H7N9 were found to elicit protective immunity against lethal challenges.

### M2 conserved domain vaccine

ACAM-FLU-A is an influenza M2 ectodomain vaccine developed by Acambis (now Sanofi Pasteur). Due to overlapping nucleotides with M1, the M2 ectodomain is highly conserved in influenza A viruses, but poorly immunogenic [74]. ACAM-FLU-A utilizes the Hepatitis B core (HBc) as a carrier to fuse three tandem repeats of the M2 ectodomain onto each HBc subunit, creating an immunogenic virus-like particle (VLP). Initial results showed intramuscular injection of the vaccine was able to generate anti-M2 ectodomain seroconversion in 90% of healthy volunteers [73]. However, after immunization M2-specific antibody titers steadily declined over a 1-year period [86], so combination with the other antigens or adjuvants might be necessary.

## Challenges for universal influenza vaccine development

### The need for accurate surrogate markers of VE for clinical study and licensing approval

Precisely characterizing influenza immunity and correlates of immune protection is one of the three major areas for improvement outlined in NIAID’s strategic plan for a universal influenza vaccine [87]. Serological assays such as hemagglutination inhibition (HAI) and single radial hemolysis (SRH) have long been held by regulatory agencies as a correlate of protection for inactivated influenza vaccine licensure. European Medicines Agency’s (EMA) Committee for Human Medicinal Products (CHMP) criteria indicates that for seasonal influenza vaccine approval one of three conditions must be met: seroprotection (HI titer of ≧1:40 or SRH of 25 mm^2^) rate of over 70%, seroconversion (4-fold increase in titer) rate more than 40%, or a geometric mean increase (pre- vs post-vaccination) of 2.5 times in healthy adults, and 60, 30%, 2.0x respectively for elders [88]. The US FDA Center for Biologics Evaluation and Research (CBER) follows a similar criterion for accelerated approval [89].

However, HAI and SRH assays may not always be applicable when it comes to LAIV or novel next-generation vaccines currently under development. HAI measures the antibody-mediated inhibition of erythrocyte agglutination caused by HA binding to sialic acids on the erythrocyte surface. As such, the assay only detects antibodies directed at the HA head domain where its receptor binding site is located. Universal vaccine strategies based on eliciting immune response against conserved epitopes on the HA stem domain, M2, M1 or NP would not be detected by the HAI assay. SRH detects the concentration of influenza-targeting antibodies by measuring a ring of hemolysis caused by the antibody-virus-erythrocyte complex activating the complement system [90]. While this method measures all serum antibodies against influenza surface antigens, it still does not recognize local mucosal immunity or cell-mediated immunity, such as immunization strategies that target M1 or NP [91].

This has led to the recognition that non-HAI or SRH assays need to be taken into account for regulatory approval of next-generation influenza vaccines [87, 88], though challenges in standardization of assays and reproducibility between laboratories still need to be overcome. Finally, human challenge trials are gaining acceptance by regulatory agencies for universal vaccine development which may lack traditional serological correlates for protection [87, 92–94]. There is increasing recognition that utilizing all aspects of our immune system are needed to control influenza outbreaks.

### Eligibility for vulnerable groups

Elderly people often have more serious complications from influenza infections and a less robust immune response to vaccination [95]. Currently, high dose or adjuvanted IIVs are recommended for people 65 years and older, while LAIV is only approved for healthy adults up to the age of 49. On the other end of the spectrum, maternally-derived antibodies generated from inoculation during pregnancy are expected to provide protection for infants < 6 months, so vaccination that elicit a predominantly cell-mediated immune response are unlikely to be of use. Novel strategies for a universal flu vaccine will have to take into account differences in immune response from specific populations that are at higher risk for influenza complications.

### Long-term protection

With traditional seasonal flu vaccine human immunity wanes in 6–8 months of time, enough to last through the influenza season [96, 97]. But if a universal vaccine were to break the cycle of yearly vaccinations, long-term protection will be needed. Having durable protection for at least 1 year and preferably through multiple seasons is one of the four criteria set by the NIAID for a universal influenza vaccine [87], but how to achieve that goal is still unknown. Immunization schedules, formulations, dosages, and adjuvants will all likely have to be considered.

## Conclusions

The evolution of influenza vaccine development has shown a trend of cell-based vaccines gradually taking the place of traditional egg-based manufacture. With the plethora of next-generation vaccines currently under development, WHO expects a universal influenza A vaccine to be in advanced clinical trials as early as 2027 [98]. Although many candidates have shown promising results in preclinical studies, demonstrating clinical safety and efficacy in a human population remains the most significant hurdle towards regulatory approval.

Our group has pioneered the strategy of exposing previously-shielded conserved epitopes on the HA through enzymatic trimming of N-glycans. This technique has been shown to elicit cross-neutralizing antibodies against antigenically diverse strains of influenza viruses within a subtype [52, 53], and thus hypothetically a trivalent or tetravalent monoglycosylated vaccine containing the three influenza subtypes (H1, H3, and influenza B) circulating in the human population would be, for all intents and purposes, a universal flu vaccine.

We believe this monoglycosylated split virus vaccine strategy has three unique qualities that give it a significant advantage in the new drug developmental process:

### The monoglycosylated split vaccine provides multiple conserved epitopes for immune recognition

Due to the rapid mutation rate of the influenza virus, using only a single conserved epitope as the antigenic target for universal vaccine runs the risk of generating escape mutants [99, 100]. In our previous studies we have only demonstrated the concept that monoglycosylated split virus vaccine induces more stem-specific antibodies directed against conserved epitopes on the HA stem [54]. However, in theory by trimming off oligosaccharides on every N-glycosylation site on the HA, multiple conserved epitopes would be exposed, inducing a multi-faceted immune response that imposes a higher evolutionary barrier for escape mutant generation. Another influenza glycoprotein that could potentially benefit from the monoglycosylation process is NA. The preparation of monoglycosylated split virus vaccine would remove glycans from not only HA but also NA, hypothetically inducing more anti-NA antibodies that interfere with virus budding, disease progression and severity of symptoms [101].

### The monoglycosylated split vaccine induces a similar immune response to current IIVs, meeting established surrogates of VE

Although a more diversified criteria encompassing CMI, neutralization assays and NA antibodies is encouraged, traditional serological assays remain the gold standard for regulatory approval. By incorporating our monoglycosylation technology onto the existing inactivated split vaccine platform, we could invoke a similar humoral response as conventional IIVs. Serological surrogates of vaccine efficacy such as HAI or SRH can be measured and non-inferiority comparisons with conventional vaccines can be made, opening up a well-trodden path towards licensure.

### The monoglycosylated split vaccine is suitable for all age groups

Whether novel vaccine strategies that are effective on healthy adults are equally suitable for all age groups remains a concern. Due to having the same constituents as an IIV, the monoglycosylated split vaccine can be expected to offer a similar safety profile as the conventional flu vaccine. As such, it is possible that formulations suitable for different age groups, such as reduced dosage for children and high dose/adjuvanted vaccines for the elderly, can also be applied to our monoglycosylated split vaccine. Furthermore, the robust humoral immunity induced by IIV assures sufficient protection for infants < 6 months by maternal vaccination.

Even though recent advances in influenza vaccine manufacture such as cell-based and recombinant HA have allowed for a much quicker production timeline, using conventional strain-specific vaccines against a rapidly evolving influenza virus assures we are always playing catch-up. As our understanding of influenza pathogenesis and immune response continues to grow, developing a universal vaccine that provides long-lasting protection against divergent strains or subtypes is becoming an increasingly attainable goal. We believe our monoglycosylated split vaccine strategy that applies a simple modification step to pre-existing egg-based production platforms to provide broader immunity in the end product, is a significant step towards this goal.

## Data Availability

Not applicable.

## Abbreviations

ADCC: Antibody-dependent cellular cytotoxicity
bnAbs: broadly neutralizing Antibodies
CBER: Center for Biologics Evaluation and Research
CDC: Centers for Disease Control and Prevention
CHMP: Committee for Human Medicinal Products
CMI: Cell Mediated Immunity
CVVs: Candidate Vaccine Virus
EMA: European Medicines Agency
FDA: Food and Drug Administration
GISRS: Global Influenza Surveillance and Response System
GlcNAc: N-Acetylglucosamine
GnTI^−^: N-acetylglucosaminyltransferase I ^−^
HA: Hemagglutinin
HA_fg_: fully glycosylated HA
HAI: Hemagglutination Inhibition
HA_mg_: Monoglycosyalted HA
HBc: Hepatitis B core
IIV: Inactivated Influenza Vaccine
IIV3-HD: high-dose inactivated trivalent influenza vaccine
LAIV: Live attenuated influenza vaccine
M1: Matrix 1 protein
M2: Matrix 2 protein
M2SR: M2 knockout vaccine
MDCK: Madin-Darby Canine Kidney
MN: Microneutralization
NA: Neuraminidase
NIAID: National Institute of Allergy and Infectious Diseases
NP: Nucleoprotein
RdRP: RNA-dependent RNA polymerase
SRH: Single Radial Hemolysis
tNIV: trivalent nanoparticle influenza vaccine
VE: Vaccine Effectiveness
WHO: World Health Organization
